# Application value of a deep learning method based on a 3D V-Net convolutional neural network in the recognition and segmentation of the auditory ossicles

**DOI:** 10.3389/fninf.2022.937891

**Published:** 2022-08-31

**Authors:** Xing-Rui Wang, Xi Ma, Liu-Xu Jin, Yan-Jun Gao, Yong-Jie Xue, Jing-Long Li, Wei-Xian Bai, Miao-Fei Han, Qing Zhou, Feng Shi, Jing Wang

**Affiliations:** ^1^Xi’an Key Laboratory of Cardiovascular and Cerebrovascular Diseases, Xi’an No.3 Hospital, Affiliated Hospital of Northwest University, Xi’an, China; ^2^Department of Research and Development, Shanghai United Imaging Intelligence Co., Ltd., Shanghai, China; ^3^Department of Medical Imaging, Xi’an Hospital of Traditional Chinese Medicine, Xi’an, China

**Keywords:** auditory ossicles, automatic segmentation, computed tomography, convolutional neural network, deep learning

## Abstract

**Objective:**

To explore the feasibility of a deep learning three-dimensional (3D) V-Net convolutional neural network to construct high-resolution computed tomography (HRCT)-based auditory ossicle structure recognition and segmentation models.

**Methods:**

The temporal bone HRCT images of 158 patients were collected retrospectively, and the malleus, incus, and stapes were manually segmented. The 3D V-Net and U-Net convolutional neural networks were selected as the deep learning methods for segmenting the auditory ossicles. The temporal bone images were randomized into a training set (126 cases), a test set (16 cases), and a validation set (16 cases). Taking the results of manual segmentation as a control, the segmentation results of each model were compared.

**Results:**

The Dice similarity coefficients (DSCs) of the malleus, incus, and stapes, which were automatically segmented with a 3D V-Net convolutional neural network and manually segmented from the HRCT images, were 0.920 ± 0.014, 0.925 ± 0.014, and 0.835 ± 0.035, respectively. The average surface distance (ASD) was 0.257 ± 0.054, 0.236 ± 0.047, and 0.258 ± 0.077, respectively. The Hausdorff distance (HD) 95 was 1.016 ± 0.080, 1.000 ± 0.000, and 1.027 ± 0.102, respectively. The DSCs of the malleus, incus, and stapes, which were automatically segmented using the 3D U-Net convolutional neural network and manually segmented from the HRCT images, were 0.876 ± 0.025, 0.889 ± 0.023, and 0.758 ± 0.044, respectively. The ASD was 0.439 ± 0.208, 0.361 ± 0.077, and 0.433 ± 0.108, respectively. The HD 95 was 1.361 ± 0.872, 1.174 ± 0.350, and 1.455 ± 0.618, respectively. As these results demonstrated, there was a statistically significant difference between the two groups (*P* < 0.001).

**Conclusion:**

The 3D V-Net convolutional neural network yielded automatic recognition and segmentation of the auditory ossicles and produced similar accuracy to manual segmentation results.

## Introduction

**FIGURE 1:**
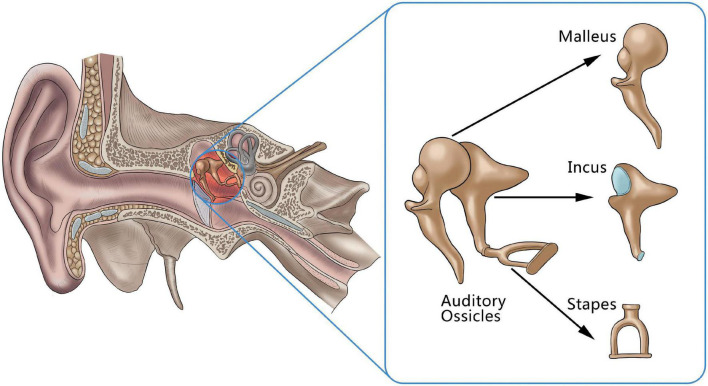
An anatomical illustration of auditory ossicles located in the middle ear, including the malleus, incus, and stapes.

Hearing impairment caused by lesions of the auditory ossicles is a common clinical disease, and both the destruction of the auditory ossicles caused by inflammation and tumors and malformation or dysplasia of the auditory ossicles can lead to hearing impairments, and even to hearing loss (Juliano et al., 2013, 2015). The ossicle chain, which comprised the three auditory ossicles (the malleus, incus, and stapes), acts as a mechanism for conducting sound in the middle ear (Figure 1). As its structure is small and fine, it can be difficult for clinicians to find abnormal auditory ossicles by physical means, making an auditory ossicle reconstruction operation difficult and requiring high-risk technology. However, accurate auditory ossicle evaluation is important for the diagnosis and pre-treatment evaluation of patients with hearing impairment. Therefore, it is critical to correctly master the anatomy of deformed auditory ossicles to formulate a surgery plan.

High-resolution computed tomography (HRCT) of the temporal bone can achieve submillimeter resolution and is the established standard for the clinical detection of anatomical abnormalities of the temporal bone in patients with ear disease (Jäger et al., 2005). In addition to clarifying the location and degree of the lesions, it can play a decisive role in evaluating illnesses, such as auditory ossicle malformation, trauma, inflammation, and temporal bone tumor; it can also provide a reliable basis for understanding the location and scope of the lesions, selecting surgery methods, and improving the safety of these methods (Tatlipinar et al., 2012).

The morphological characteristics of the auditory ossicles are accurately displayed by HRCT. However, the structure of the auditory ossicles is quite small; the total length of an adult stapes is approximately 3 mm (Noussios et al., 2016). In addition, not all parts of the auditory ossicles can be displayed on the same level in conventional transverse axial CT images. It can thus be challenging to accurately evaluate them using conventional axial images.

In clinical practice, the entire image can be displayed only after post-processing reconstruction of a curved planar reconstruction (CPR), multiplanar reformation (MPR), and volume rendering (VR). This post-processing reconstruction process is time-consuming and requires a higher level of expertise and experience from ear, nose, and throat (ENT) radiologists, which may result in reduced work efficiency and an increase in missed diagnoses or misdiagnoses.

With the maturity of computer-aided diagnosis technology, medical imaging artificial intelligence (AI)-aided diagnosis equipment has been widely used to study several organ structures (e.g., the lung, liver, breast, and bone) (Hosny et al., 2018). As an important branch of AI, deep learning technology, which has made significant progress since 2012, has been widely used in image classification (He et al., 2020), lesion detection, and segmentation (Ren et al., 2017; Falk et al., 2019). In some respects, it has reached or exceeded the diagnostic level of clinicians, as is the case in the diagnosis and prognosis evaluation of hepatobiliary malignant tumors (Ibragimov et al., 2018; Zhou et al., 2019), the early diagnosis and pathological classification prediction of lung cancer (Coudray et al., 2018), the establishment of a breast cancer diagnosis model to predict malignant breast cancer (Li, 2021), and the automatic detection of fundus images identifying glaucoma and diabetic retinopathy (Schmidt-Erfurth et al., 2018; Ting et al., 2019).

Despite these technological advancements, few mature studies exist on the AI-aided diagnosis of smaller structures, e.g., the auditory ossicles. This study aims to explore a computer segmentation and recognition technology of auditory ossicles, based on HRCT images, which could assist radiologists in making more accurate evaluations and diagnoses of potential auditory ossicle abnormalities, such as destruction, absence, malformation, or dysplasia.

## Materials and methods

### Data and structure labeling

The 158 samples used in this study were from adult patients (aged older than 18 years, mean age, 38.60 ± 18.05), who underwent temporal bone CT scanning in Xi’an Central Hospital and Xi’an No.3 Hospital from 2015 to 2019. Senior ENT radiologists confirmed the absence of any structural abnormalities of the external ear, middle ear, or inner ear from the samples provided. A Philips Brilliance iCT 256 was used for scanning using the following settings: scanning parameters, 120 kV, 200 mAs; rotation speed 0.4 s/360°; collimation, 0.625 mm; field of view, 250 mm × 250 mm; reconstruction matrix, 1024 × 1024; reconstruction interval, 0.2–0.3 mm; bone algorithm reconstruction. All scans were made from the lower edge of the external auditory canals to the upper edge of the petrous bones, with a scanning length of 1.5 cm. The samples were divided into three groups as follows: 80% were randomly selected from the data set as the training set, including the CT data of 126 cases of unilateral normal temporal bone images; 10% were selected as the test set, including the CT data of 16 cases of unilateral normal temporal bone images; 10% were selected as the validation set, including the CT data of 16 cases of unilateral normal temporal bone images.

Manual labeling of the auditory ossicle structure was conducted as follows: the original DICOM data of the HRCT images of the temporal bones were imported into ITK-SNAP 3.2, and the malleus, incus, and stapes were manually labeled under the “bone” window. The labeling process was performed by two ENT radiologists. One radiologist completed the specific segmentation and the second performed the review.

### Method

#### Model establishment and implementation of the deep learning method

**FIGURE 2:**
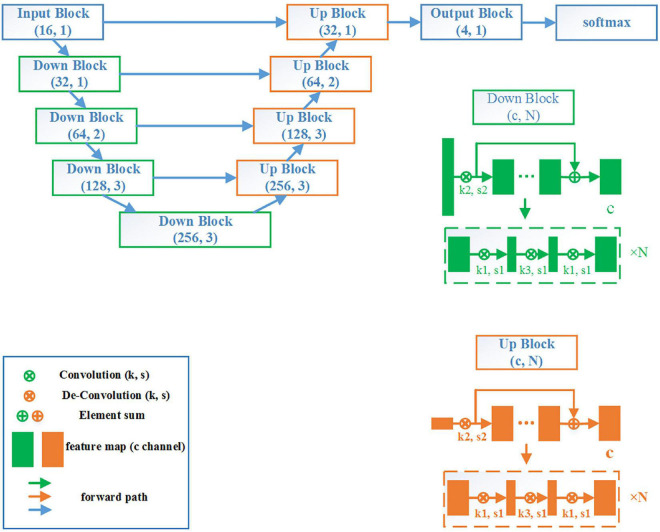
The VB-Net network structure.

The neural network applied in this study was constructed as follows: two cascaded neural networks (from low to high resolution) were adopted as shown in Figure 2. The network structure was combined with the bottleneck structure, based on the V-Net backbone. Due to using the bottleneck structure, the network was labeled a VB-Net (“B” denoting “bottleneck”), which reduced the network parameters and increased the network depth. As shown in Figure 2, the VB-Net comprised one input block, four down blocks, four up blocks, one output block, and one Softmax module. In each block, the channel of the output feature map and the number of bottlenecks is shown in the brackets, respectively. The down block comprises one convolution module and some bottlenecks, and the up block includes one de-convolution module and some bottlenecks. The kernel and stride size of the convolution and de-convolution modules are set as k and s, respectively. The bottleneck structure is illustrated in the dotted rectangular box.

**FIGURE 3:**
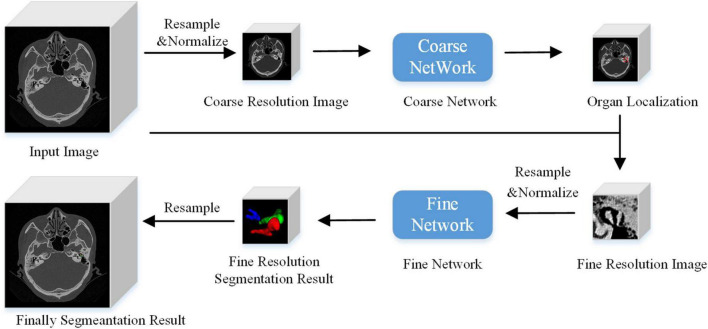
Algorithm flow chart.

The algorithm flow chart is shown in Figure 3. At a low image resolution, the general positions of the malleus, incus, and stapes could initially be located. Then, the edges of the three auditory ossicles were finely segmented at a relatively high image resolution.

##### Model training

The two segmentation tasks concerning the auditory ossicles and the image background were trained at a low resolution. First, the outline markers of the malleus, incus, and stapes were combined, and a three-dimensional (3D) morphological expansion algorithm was adopted with a 1 cm expansion in each direction as the ground truth of the coarse locating model. Then, the HRCT image was resampled to [1, 1, 1 mm], a point was randomly selected on the entire image, and an image block of [96, 96, 64] was cut out using this point as the center area. This image block was input into the 3D VB-Net for low-resolution locating model training. The window level of the image normalization was 1,000 HU, and the window width was 3,000 HU. The intensity value, with a CT value between –2,000 and 4,000 HU, was linearly normalized to [–1, 1]. The intensity values greater than 4,000 HU were normalized to 1, and those below –2,000 HU were normalized to –1. A single-class Dice loss function and a cross-entropy loss function were selected as the loss functions, but the weights of both loss functions were equal.

At a high resolution, the malleus, incus, stapes, and the background were segmented. The HRCT image was resampled to [0.2, 0.2, 0.335 mm]. Then, a random point was selected within 20 pixels of the malleus, incus, and stapes masks as the center point to cut out a [96, 96, 96] image block, which was subsequently input into the 3D VB-Net for high-resolution fine segmentation model training. The normalization method was the same as that of the coarse-resolution model training noted above. The loss functions were a multi-class Dice and a cross-entropy loss function, respectively, but the weights of both loss functions were equal. When calculating the loss function, the weights of each class were equal.

##### Model reasoning

Unlike the image block input in the training stage, the test stage was subject to the network reasoning of the entire image. A multi-resolution strategy was used to connect the low- and high-resolution networks. The goal of the low-resolution network was to roughly locate the position of the organ (the segmentation target: the malleus, incus, and stapes). The entire image was resampled to an isotropic spacing of 1 mm, and the network only focused on the overall region of interest of the auditory ossicles at a lower image resolution. At a high resolution, only the region of interest (extended by 2 cm), obtained from the low-resolution network, was resampled and input into the trained high-resolution network to create the accurate segmentation of each organ boundary under a fine image resolution. A combination of coarse and fine resolutions was adopted for the post-processing of the model.

#### Segmentation accuracy test

This study verified the stability and effectiveness of the presented model using five-fold cross-validation. That is, 158 sample data sets were randomly divided into a training set, a validation set, and a test set according to an 8:1:1 ratio, and the average accuracy of the model was obtained by averaging the five-fold network’s accuracy. The manual segmentation results of the ENT radiologist served as the ground truth (Powell et al., 2017), and the 3D V-Net and a 3D U-Net model were employed. The following evaluation indicators were used to measure the performance of the segmentation method in this study (Taha and Hanbury, 2015). The dice similarity coefficient (DSC), with a value range from 0 to 1, was used to reflect the similarity coefficient between the automatic and manual segmentation. A value closer to 1 indicated a better model. When the DSC was more than or equal to 0.7, the automatic and manual segmentations were considered to indicate good consistency (Zhong et al., 2021). The DSC is defined as,


$$D\,S\,C=\frac{2^{*}\,\left(R\,p*R\,t\right)}{R\,p+R\,t}*100\%$$

where *R_t_* and *R_p_* are denoted as the gold mask and the predicted mask, respectively.

The average surface distance (ASD) referred to the average deviation of the surface distance of all points on the average surface. The ASD is defined as,


$$A\,S\,D\,\left(X,Y\right)=\sum_{x\in X}m\,i\,n_{y\in Y}\,d\,\left(x,y\right)/\left|X\right|$$

where *X* and *Y* are the set of points on the boundary of *R_t_* and *R_p_*, respectively. *d*(*x*, *y*) is denoted as the Euclidean distances between two points.

The maximum Hausdorff distance is defined as,


$$H\,D\,\left(X,Y\right)=\max_{x\in X}\left\{\min_{y\in Y}\left\{d\,\left(x,y\right)\right\}\right\}$$

where *X* and *Y* are the set of points on the boundary of *R_t_* and *R_p_*, respectively. *d*(*x*, *y*) is denoted as the Euclidean distances between two points.

The HD95 is the 95th percentile of Hausdorff distance set.

#### Statistical method

The SPSS Statistics 22.0 (IBM Company, Armonk, NY, United States) software program was used to conduct paired *t*-tests on the measured values that were obtained by different methods; *P* < 0.05 indicated a statistically significant difference.

#### Implementation details

The Adam optimizer (initial learning rate = 0.01) algorithm was chosen to minimize the loss of neural network. The specific loss of dice is adopted in our method. The dice loss function is defined as,


$$L_{D\,i\,c\,e}=1-\frac{2*\left(R_{p}*R_{t}\right)}{R_{p}+R_{t}}*100\%$$

where *R_t_* and *R_p_* are denoted as the gold mask and the predicted mask, respectively.

The optimal training epoch of each model was selected, based on the minimum loss of validation data, and the training process was considered to have converged if the loss stopped decreasing for 20 epochs. The framework was implemented in PyTorch 1.7.0 with one Nvidia Tesla V100 graphics processing unit.

## Results

**FIGURE 4:**
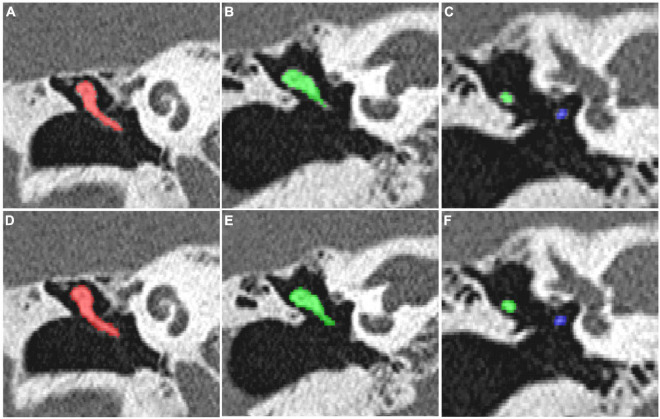
**(A–C)** The manual segmentation of the auditory ossicles; **(D–F)** the automatic segmentation of the auditory ossicles using the V-Net method.

For the CT scan of the temporal bone, the ITK-SNAP 3.2 software was used to manually segment all the auditory ossicles, and the manual annotation of the malleus, incus, and stapes were used for the automatic segmentation training of the 3D V-Net and U-Net network structures. The network parameters were obtained through data learning, and the comparison between segmentation of model and expert is shown in Figure 4.

As listed in Table 1, patch size and spacing were effective parameters for the model’s performance. To select suitable experimental configurations, the model’s performance, using different training configurations, was compared in accordance with the DSC, the ASD, and the HD 95 (see Table 2). Table 2 indicates that in the coarse model training configuration, the best patch size and spacing were [96, 96, 64] and [1, 1, 1] mm, respectively. The best patch-size and spacing values in the fine model training configuration were [96, 96, 96] and [0.2, 0.2, 0.335] mm, respectively. According to the comparison experiments, the following performance was achieved by the 3D V-Net and 3D U-Net networks using the above-noted configurations.

**TABLE 1:** The detailed configuration for coarse-to-fine segmentation network.

Configuration	Coarse network	Fine network
Resample	[1, 1, 1] mm	[0.2, 0.2, 0.335] mm
Patch size	[96, 96, 64]	[96, 96, 96]
Normalize	*z*-score with fixed mean (1000) and standard (3000/2) and clipping to [–1, 1]	*z*-score with fixed mean (1000) and standard (3000/2) and clipping to [–1, 1]
Learning rate	Step learning rate schedule (initial, 1e-2)	Step learning rate schedule (initial, 1e-2)
Optimizer	Adam [momentum = 0.9, decay = 1e-4, betas = (0.9, 0.999)]	Adam [momentum = 0.9, decay = 1e-4, betas = (0.9, 0.999)]
Software	PyTorch	PyTorch
Hardware	Nvidia Tesla V100 GPU	Nvidia Tesla V100 GPU

**TABLE 2:** Comparison of segmentation performance between different experiment settings (patch size, patch spacing) under 3D V-Net.

	DSC		
	
	Malleus	Incus	Stapes
Es1	0.800 ± 0.018	0.787 ± 0.051	0.528 ± 0.103
Es2	**0.920 ± 0.014**	**0.925 ± 0.014**	**0.835 ± 0.035**
Es3	0.836 ± 0.242	0.846 ± 0.237	0.799 ± 0.236
Es4	0.809 ± 0.048	0.797 ± 0.057	0.609 ± 0.102
Es5	0.817 ± 0.045	0.797 ± 0.059	0.618 ± 0.103

	**ASD**		
	
	**Malleus**	**Incus**	**Stapes**

Es1	0.654 ± 0.047	0.642 ± 0.040	0.580 ± 0.108
Es2	**0.257 ± 0.054**	**0.236 ± 0.047**	**0.258 ± 0.077**
Es3	28.990 ± 110.4	31.8 ± 120.1	26.2 ± 102.4
Es4	0.636 ± 0.170	0.700 ± 0.210	0.649 ± 0.168
Es5	0.638 ± 0.170	0.706 ± 0.217	0.652 ± 0.168

	**HD95**		
	
	**Malleus**	**Incus**	**Stapes**

Es1	1.439 ± 0.170	1.594 ± 0.664	4.393 ± 2.368
Es2	**1.016 ± 0.080**	**1.000 ± 0.000**	**1.027 ± 0.102**
Es3	31.3 ± 113.7	33.9 ± 123.6	28.2 ± 104.7
Es4	1.740 ± 1.965	1.332 ± 0.188	1.475 ± 0.0383
Es5	1.332 ± 0.236	1.351 ± 0.208	1.334 ± 0.237

Es1: In the coarse model training configuration, patch size and spacing were set as [96, 96, 64] and [2.5, 2.5, 2.5] mm, respectively. In the fine model training configuration, patch size and spacing were set as [96, 96, 96] and [0.5, 0.5, 0.75] mm, respectively.

Es2: In the coarse model training configuration, patch size and spacing were set as [96, 96, 64] and [1, 1, 1] mm, respectively. In the fine model training configuration, patch size and spacing were set as [96, 96, 96] and [0.2, 0.2, 0.335] mm, respectively.

Es3: In the coarse model training configuration, patch size and spacing were set as [96, 96, 64] and [0.5, 0.5, 0.5] mm, respectively. In the fine model training configuration, patch size and spacing were set as [96, 96, 96] and [0.1, 0.1, 0.2] mm, respectively.

Es4: In the coarse model training configuration, patch size and spacing were set as [128, 128, 96] and [1, 1, 1] mm, respectively. In the fine model training configuration, patch size and spacing were set as [128, 128, 128] and [0.2, 0.2, 0.335] mm, respectively.

Es5: In the coarse model training configuration, patch size and spacing were set as [64, 64, 48] and [1, 1, 1] mm, respectively. In the fine model training configuration, patch size and spacing were set as [64, 64, 64] and [0.2, 0.2, 0.335] mm, respectively.

Bold fonts represent the best performance among all training configurations.

On completion of the V-Net network structure training, the auditory ossicles and the manual segmentation images were compared for accuracy using the test set and evaluated in accordance with the DSC, the ASD, and the HD 95. The specific results are shown in Tables 3–5. Compared with the U-Net method, the 3D V-Net method delivered a segmentation effect similar to that of manual segmentation, and the difference was statistically significant.

**TABLE 3:** Comparison of DSC between automatic segmentation and manual segmentation under two neural networks.

Network structure	DSC		
	
	Malleus	Incus	Stapes
3D V-Net	0.920 ± 0.014	0.925 ± 0.014	0.835 ± 0.035
U-Net	0.876 ± 0.025	0.889 ± 0.023	0.758 ± 0.044
*T*	–13.602	–11.762	–11.727
*P*	< 0.001	< 0.001	< 0.001

**TABLE 4:** Comparison of ASD between automatic segmentation and manual segmentation under two neural networks.

Network structure	ASD		
	
	Malleus	Incus	Stapes
3D V-Net	0.257 ± 0.054	0.236 ± 0.047	0.258 ± 0.077
U-Net	0.439 ± 0.208	0.361 ± 0.076	0.433 ± 0.108
*T*	7.4500	12.1940	11.965
*P*	< 0.001	< 0.001	< 0.001

**TABLE 5:** Comparison of HD95 between automatic segmentation and manual segmentation under two neural networks.

Network structure	HD95		
	
	Malleus	Incus	Stapes
3D V-Net	1.016 ± 0.080	1.000 ± 0.000	1.027 ± 0.102
U-Net	1.361 ± 0.872	1.174 ± 0.350	1.455 ± 0.618
*T*	3.4559	4.3487	5.4810
*P*	< 0.001	< 0.001	< 0.001

**FIGURE 5:**
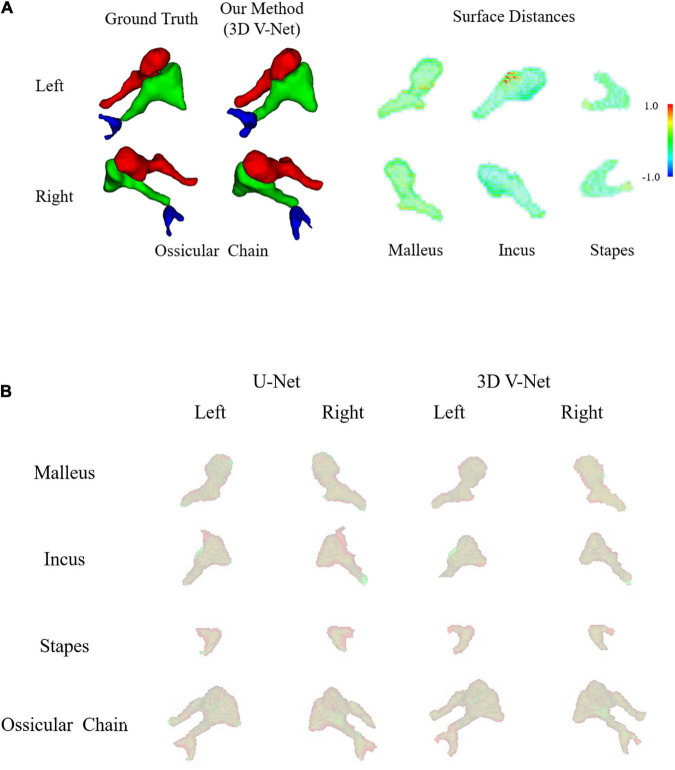
**(A)** A three-dimensional display of the segmentation results. On the left side is the segmentation result of the ground truth and the 3D V-Net method. The right side shows the difference in the surface distance between the two results. The auditory ossicles comprise the malleus, incus, and stapes. In this paper, positive values represent over-segmentation, and negative values represent under-segmentation. The left side of the figure corresponds to the left auditory ossicles, and the right corresponds to the right auditory ossicles. **(B)** Comparison of the segmentation results. The segmentation results of the two methods are compared with the surface coincidence degree of the ground truth. Green is the ground truth reconstruction, and red is the segmentation result reconstruction.

The 3D display of the segmentation result is shown in Figure 5A. The left area of the figure shows the entire auditory ossicle structure, while the right side shows the malleus, incus, and stapes, respectively. Compared with the ground truth, the result of the method was consistent and showed little difference concerning surface distance. Figure 5B shows a comparison of the results of the U-Net and 3D V-Net methods. The result of the U-Net method shows a significant difference between the (green) ground truth reconstruction and the (red) segment reconstruction result.

The image segmentation time evaluation proceeded as follows. The average daily work time required for two senior physicians, two intermediate physicians, two junior physicians, and two resident physicians to manually segment 50 cases of auditory ossicles was counted. The average time required for these eight physicians to manually segment the ossicles was 220.31–387.42 s, and the average time required for the model to automatically segment the ossicles was 1.66 s (0.83 s required to segment the left and right ossicles). These results are shown in Table 6.

**TABLE 6:** Average time for manual segmentation and model segmentation of auditory ossicles.

	Average segmentation time/second (s)
Senior Physician 1	220.31
Senior Physician 2	225.64
Intermediate Physician 1	253.53
Intermediate Physician 2	258.12
Junior Physician 1	300.78
Junior Physician 2	310.56
Resident Physician 1	375.31
Resident Physician 2	387.42
3D U-Net	2.00
3D V-Net	1.66

## Discussion

### The clinical practice significance of high resolution computed tomography ossicular chain segmentation

The ossicular chain, comprising the malleus, incus, and stapes, is deeply situated within the ear and covered by the tympanic membrane. It acts as a device for conducting sound in the middle ear, and changes in the morphology, position, and density of each auditory ossicle may cause hearing disorders. An accurate evaluation of the auditory ossicles is important for the diagnosis and pretherapeutic evaluation of patients experiencing hearing loss. However, the ossicular chain is situated deep inside the ear, small and delicate, and making a diagnosis related to it is often challenging.

High-resolution computed tomography is an indispensable examination method because it can be used to accurately and clearly reflect the structure of the auditory ossicles (Hiraumi et al., 2019). However, the auditory ossicles are small and are not all located on the same plane. Therefore, it is difficult to observe the auditory ossicles on a conventional CT axial image. Additionally, radiologists must thoroughly observe hundreds of images and conduct post-processing reconstruction of the auditory ossicles (i.e., MPR, CPR, and VR) to evaluate them, which relies heavily on the clinical experience and proficiency of the radiologist as it concerns post-processing techniques and is a time-intensive process that is accompanied by a probability of misdiagnosis or missed diagnosis.

Although the AI-assisted diagnosis technique has been widely applied, research on the AI-assisted diagnosis of small-scale structures, such as the auditory ossicles, is rare. The present study adopted HRCT-based AI to create fully automatic segmentation of the auditory ossicles, thereby contributing to satisfying the needs of patients with ear diseases. Automatic identification and segmentation of the auditory ossicles can significantly assist radiologists and clinicians in making an accurate diagnosis.

### Comparison between the artificial intelligence algorithm and other algorithms, and a summary of its advantages and disadvantages

The technical method of computer segmentation of the auditory ossicle structure in the research literature is mainly based on mapping, neural networks, and deep learning. The Powell algorithm is used to automatically segment the anatomical structure in CT images of the temporal bone based on anatomical atlas. The results indicated that the DSC of the malleus and incus on both sides was greater than 0.80, and the DSC of the left and right stapes was 0.58 and 0.48 (Powell et al., 2017), respectively. Mapping-based automatic segmentation delivers excellent results in the normal anatomical structure, but the registration accuracy limits the method, and any anatomical variation may cause the failure of automatic identification and segmentation (Noble et al., 2009; Powell et al., 2017, 2019).

The neural network and deep learning method make up for the deficiency of the above method. Currently, with respect to the neural network structure, the most common network architecture includes a fully convolutional network (FCN) (Shelhamer et al., 2017), a U-Net (Ronneberger et al., 2015), a 3D U-Net (Iek et al., 2016), and a V-Net (Milletari et al., 2016). The FCN was a pioneer of image segmentation and the deep learning technique and adopted an end-to-end convolutional neural network and deconvolution for upsampling. However, as it is not sensitive to image details and can cause a partial loss of information, its segmentation accuracy is low for small structures. Ronneberger et al. (2015) proposed a U-Net method, based on an FCN, and applied the full convolutional network to the field of medical image segmentation. However, the FCN and U-Net can only be used for the identification and segmentation of two-dimensional images, whereas a 3D U-Net and V-Net can be used for the identification and processing of 3D images. Compared to a 3D U-Net, V-Net training gradually became the primary method of medical image segmentation due to its high velocity and the short time it requires to complete (Milletari et al., 2016). Fauser adopted a U-Net method to segment the temporal bone before surgery; in this instance, the DSC of the auditory ossicles was up to 0.75 (Fauser et al., 2019). Lv et al. (2021) applied the W-Net, 3D U-Net, and V-Net methods to automatically segment the temporal bone to obtain the DSCs of the auditory ossicles, which were 0.85, 0.84, and 0.83, respectively. Li et al. (2020) adopted a 3D Deep Supervised Densely (DSD) algorithm to obtain the DSCs of the malleus and the incus, which were both 0.82; however, the stapes was not segmented. Ke et al. applied a 3D convolutional neural network to successfully realize the automatic segmentation of the labyrinth, the auditory ossicles, and the facial nerve in both conventional and abnormal temporal bone CTs and achieved excellent results (Ke et al., 2020; Ding et al., 2021; Wang et al., 2021). Other scholars applied multi-view fusion and deep learning algorithms to design an accurate segmentation of the malleus and the incus and further improved the segmentation accuracy of the stapes with an active contour-loss constraint method (Zhu et al., 2021).

In clinical practice, most auditory-related diseases only involve one or two auditory ossicles. The three auditory ossicles are typically segmented as a whole in most of the existing literature (Lv et al., 2021), which fails to satisfy the needs of a clinical diagnosis and treatment, and restricts clinical application. The size of the combined three auditory ossicles is small and, as such, the segmentation accuracy will be reduced if they are segmented separately. However, the present study’s method adopted two cascade neural networks (from a low to high resolution) and added a bottleneck structure to approximately locate the combined auditory ossicles using low image resolution, then segmenting the delicate structures with high-resolution imaging. The results of this study indicated that the model image of the auditory ossicles was full, the delicate structure displayed was clearer than in a manual sketch, and the DSC of the malleus and the incus was 0.92. The DSC of the stapes was 0.86, which met clinical standards. Meanwhile, the test result of the five-fold cross-validation test proved the stability and validity of this study’s model.

The algorithm adopted in this research significantly improved the segmentation accuracy of the auditory ossicles, particularly that of the stapes. The stapes is the smallest in volume among the auditory ossicles and the most difficult to segment. The existing research adopted different technical methods to do so, but the most DSCs of the stapes was less than that of the other two auditory ossicles. Ke et al. (2020) applied mapping to segmenting the temporal bone, and the obtained DSC of the stapes was less than 0.60. Zhu et al. (2021) adopted a 10-μm grade ear specialized CT data-based multi-view fusion algorithm and an active contour loss constraint method to improve the DSC of the stapes to 0.76. Nikan et al. (2021) carried out a PWD-3DNet algorithm to obtain the DSC of the stapes, which was 0.82, whereas the DSCs of the malleus and incus were lower than that of our approach.

The current study is the first to approximately locate the auditory ossicles, separate local images from the located part, and segment each section of the auditory ossicles using high-resolution images. The coarse positioning and fine segmenting method improved the segmentation accuracy and delivered a stapes DSC of up to 0.84 at a higher image segmentation speed. In Table 7, relevant recent studies are summarized and compared with the present study. The results show that the model of the current algorithm achieved a good performance.

**TABLE 7:** Comparison of DSC values with other related studies.

	DSC			
		
	Malleus	Incus	Stapes	
Atlas-based segmentation	0.80	0.83	0.58(L),0.48(R)	Powell et al., 2017
U-Net		0.75*		Fauser et al., 2019
3D-DSD	0.82	0.81		Li et al., 2020
W-Net		0.85*		Lv et al., 2021
3D U-Net		0.84*		Lv et al., 2021
V Net		0.83*		Lv et al., 2021
Atlas-based segmentation	0.83	0.84	0.36	Ding et al., 2021
Multi-view fusion algorithm	0.94	0.95	0.76	Zhu et al., 2021
PWD-3DNet	0.89	0.89	0.82	Nikan et al., 2021
U-Net	0.88	0.89	0.76	
**3D V-Net**	**0.92**	**0.93**	**0.84**	

DSC, Dice Similarity Coefficient; L Left; R Right. *DSC of whole ossicle chain.

Bold fonts represent the results of this study.

Time is another issue to be considered in clinical application, considering that the manual reconstruction of the auditory ossicles is time-consuming. Over 1 year, the average time to reconstruct the unilateral auditory ossicles was 4 min each time, even for an expert radiologist. However, the automatic segmentation of the auditory ossicle structure, completed by the equipment and algorithms adopted in this research, took an average time of 1.66 s, which cannot be achieved by manual segmentation. Therefore, the clinical application of this model will significantly reduce the image post-processing time for the radiologist, thereby largely improving work efficiency.

### Prospects and limitations for application of the research

This research has the following limitations: (1) only automatic segmentation of the auditory ossicles was achieved; other major anatomical structures in the temporal bone, such as the cochlea, semicircular canal, and facial nerve were not segmented, thus limiting the clinical application scope of this model; (2) only normal auditory ossicles were identified and segmented; the dysplastic temporal bone was not researched, thus limiting the application of the model in cases of malformed temporal bones in clinical application. Future research on these topics will be conducted.

The existing computer-aided diagnosis system can facilitate disease diagnosis. This study evaluated the normal anatomical structure of the auditory ossicles and achieved an initial positive effect in the automatic reconstruction of the auditory ossicles. In the future, this deep learning technique can be applied to research the absence of the auditory ossicles, the malformation of the cochlea and semicircular canals, as well as deformity of the aquaeductus Fallopii to improve ear lesion diagnosis and differential diagnosis models to achieve medical diagnosis informatization and automation. Doing so will improve the work efficiency of medical staff and lower the misdiagnosis rates of ENT diseases.

## Conclusion

This study used a deep learning method to create an automatic approach to the recognition and segmentation of the auditory ossicles. The results indicated that the 3D V-Net convolutional neural network could accomplish high-precision and high-efficiency outcomes to describe the structure of the ossicular chain. It is anticipated that this will benefit the diagnosis and treatment of selected auditory system diseases, such as ossicle destruction, ossicle absence, and ossicle malformation or dysplasia.

## Data availability statement

The datasets collected and analyzed during the current study are included in this published article, and the datasets and materials are available from the corresponding author on reasonable request.

## Ethics statement

This study protocol was approved by the Clinical Research Ethics Committee of Xi’an No.3 Hospital. Written informed consent was obtained from all of the participants.

## Author contributions

X-RW completed the major data integration and manual delineation of auditory ossicles and made a critical revision of the manuscript. XM was a major contributor to manuscript drafting. L-XJ offered ethical support and participated in the collection of part of the data. Y-JG provided the major data sets and put forward the conception of the research. Y-JX and W-XB completed data statistics and analysis. J-LL offered assistance in the integration of the data. QZ devised all figures in this article and designed the algorithm flow. M-FH and FS contributed interpretation of the data and provided technical support. JW finalized the study design and supervised the quality control of the study. All authors read and approved the final manuscript.

## Abbreviations

AI: artificial intelligence
ASD: average surface distance
CPR: curved planar reconstruction
CT: computed tomography
DSC: Dice similarity coefficient
ENT: Ear, Nose, and Throat
FCN: convolutional network
HD: Hausdorff distance
HD 95: Hausdorff distance (HD) 95
HRCT: high resolution computed tomography
MPR: multiplanar reformation
VR: volume rendering.
